# A new double graft technique in urethroplasty for complex urethral stenosis: preliminary findings

**DOI:** 10.1590/S1677-5538.IBJU.2020.1131

**Published:** 2021-02-28

**Authors:** Ubirajara Barroso, Filip Prado

**Affiliations:** 1 Universidade Federal da Bahia Hospital Universitário Professor Edgard Santos Clínica de Distúrbios do Trato Urinário SalvadorBahia Brasil Clínica de Distúrbios do Trato Urinário, Hospital Universitário Professor Edgard Santos, Universidade Federal da Bahia, Salvador, Bahia, Brasil

**Keywords:** Urethral Stricture, Mouth Mucosa, Diagnostic Techniques, Surgical

## Abstract

The management of complex urethral stenosis may involve different surgical techniques. As retraction of the graft may account for surgical failure, this risk increases in patients with more extensive stenosis requiring a graft of greater diameter. Although double grafts have already been used to maximize success in these cases, we propose a modified technique for urethroplasty with longitudinal urethral incision. The hypothesis was that this technique would increase the lumen by using only a urethral incision on the dorsal surface. Two patients presenting with recurrent urethral stenosis underwent urethroplasty using a double graft of oral mucosa that preserves the integrity of the spongy tissue and allows ventral inlay graft fixation using a midline relaxing incision in the portion of the urethra with stenosis. In both cases, the urethrocystoscopy and uroflowmetry performed after surgery showed a pervious and complacent urethra. After four and six months of follow-up, the postoperative outcomes were satisfactory for both patients. Further studies involving larger numbers of patients and long-term follow-up are required to evaluate the effectiveness of this method.

## INTRODUCTION

Urethral stenosis is a pathology that is often complex and difficult to manage. Various techniques have been developed to treat it, ranging from primary anastomosis to tissue flaps and grafts (1). Currently, the preferred tissue for grafts is the oral mucosa due to its excellent physical characteristics and because harvesting is simple, with low rates of morbidity (2). Due to the wide range of urethral stenosis, different surgical techniques can be used. Despite satisfactory results, stricture recurrence has been reported in 8.9% to 34.3% of cases (3). One of the reasons for surgical failure is retraction of the graft, which may occur in around 30% of the grafted tissue. In more extensive cases of stenosis, when the caliber of the urethra is narrower, a graft of greater diameter is required, hence the risk of restenosis is greater. Double grafts have been used to maximize success in these cases. The technique described by Palminteri et al. (4) is very interesting, however, in our view there are two drawbacks: first, the need for ventral and dorsal incisions of the urethra, and second, the absence of ventral graft fixation, which, theoretically, could reduce adhesion without contractions. Our hypothesis is that the technique could be improved using only one incision on the dorsal surface which preserves the integrity of the spongy tissue and allows the use of double graft of oral mucosa with ventral inlay graft fixation using a midline relaxing incision in the portion of the urethra with stenosis.

The objective of this paper is to describe our proposed modified technique for urethroplasty with a longitudinal urethral incision and to evaluate whether this technique would allow the lumen to be increased using only a urethral incision on the dorsal surface.

## PATIENTS AND METHODS

This paper describes the outcomes of two patients who underwent urethroplasty between November 2019 and January 2020 using a double graft of oral mucosa. In both cases, previous treatments had proved unsuccessful. Data from the patients’ medical records and exam reports were used.

The institute's internal review board approved the study protocol under reference 25746819.8.0000.049. The patients gave their consent for publication.

### Description of the surgical technique

**Figure 1:**
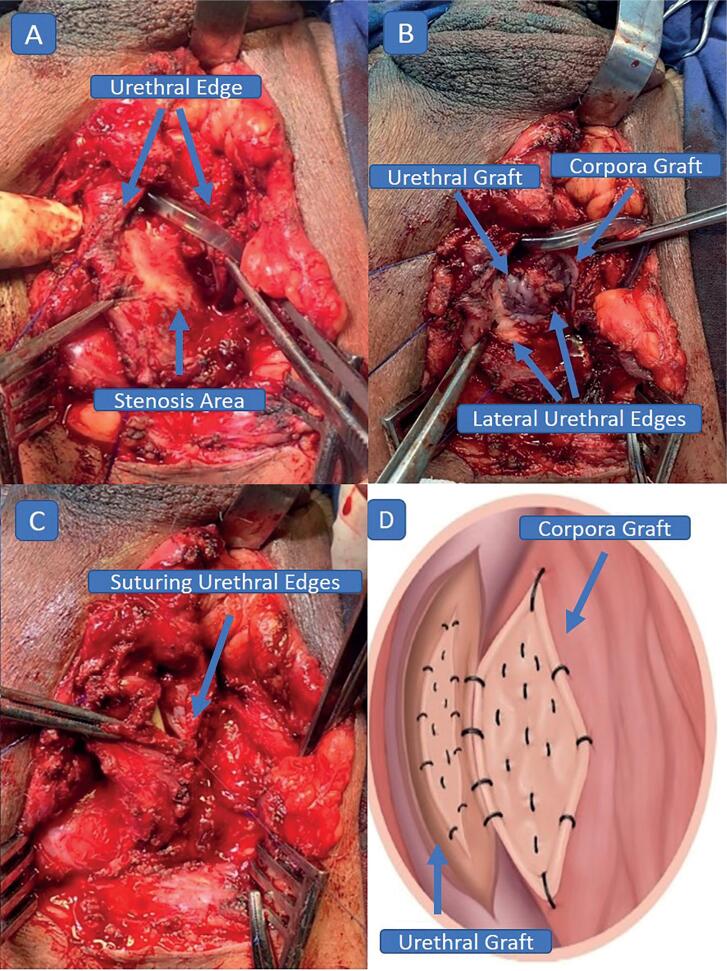
(A) Longitudinal incision in the dorsal surface of the urethra, with rotation of 180°, (B) Oral mucosa dorsal onlay and ventral inlay graft, (C) Suturing of the edges of the urethra, (D) Schematic drawing showing placement of the mucosal grafts.

The patient is placed in the exaggerated lithotomy position. A 16Fr urinary catheter is inserted, identifying the distal end of the segment affected by stenosis, and methylene blue is injected. A longitudinal incision is made in the perineum. The left and dorsal surfaces of the urethra are dissected, with the right border maintained, as proposed by Kulkarni et al. (5). However, the entire urethra can be dissected, if necessary, as described by Barbagli et al. (6). A longitudinal incision is made along the midline of the dorsal wall of the urethra affected by stenosis, extending up to 1cm beyond the area of stenosis, preserving the spongy tissue (Figure-1A). The defect created in the urethra acquires an elliptical form and the oral mucosa will be placed as an inlay graft onto this segment (Figure-1B). The incision in the urethra should proceed until the maximum degree of relaxation is achieved, preserving the corpus spongiosum. The graft is secured to the corpus spongiosum, as is usual with these grafts, using separate oral mucosa/corpus spongiosum sutures with 5-0 polydioxanone (PDS) monofilament. Next, the edge of the graft is fixed to the edge of the urethra using continuous sutures with 6-0 PDS. The remaining portion of oral mucosa is fixed onto the corpus cavernosum, as described by Barbagli et al. (6). The edges of the urethra are then sutured to the dorsal oral mucosa using 5-0 PDS suture (Figure-1C). A schematic drawing is provided to further illustrate the placement of the mucosal grafts (Figure-1D).

## RESULTS

**Figure 2:**
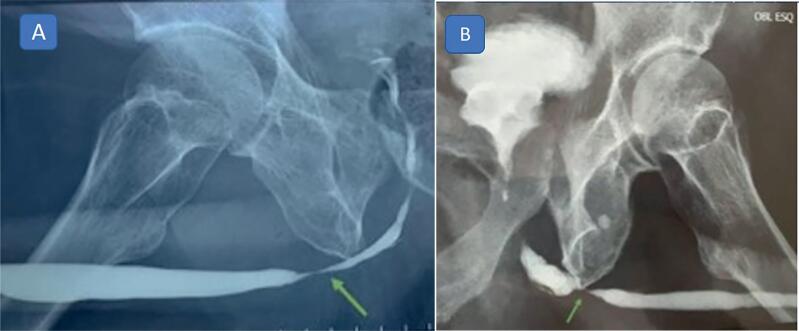
Voiding cystourethrogram: (A) Case 1 - Male, 67-year old, with a history of straddle injury to the urethra presented with penobulbar urethral stricture, (B) Case 2 - Male, 59-year old, with a history of symptomatic benign prostatic hyperplasia was submitted to transurethral resection of the prostate twice, presenting recurrent bulbar urethral stricture.

A 67-year old man with a history of straddle injury to the urethra thirty years previously presented with penobulbar urethral stricture. He had been submitted unsuccessfully to urethroplasty eight years previously, and internal urethrotomy had been performed on several occasions, as well as transurethral resection of the prostate five years previously. The patient had undergone cystostomy due to acute urinary retention a year ago and subsequently experienced several episodes of urinary tract infection. A voiding cystourethrogram was performed, with results showing stenosis from the membranous urethra to the bulbar and prostatic urethra (Figure-2A). The patient was then submitted to the surgical technique described in this paper. During surgery, the stenosis was found to be 5cm in length. Oral mucosa graft tissue of 12cm in length was harvested and divided in two for the double graft. The urinary catheter and cystotomy were maintained. The patient progressed well following surgery, with no complications. The wound in the patient's mouth healed well and the bladder catheter and cystostomy were removed 21 days after surgery. Urethrocystoscopy performed three months after surgery showed a pervious and complacent urethra. A 21Fr cystoscope was inserted with no resistance. The lateral lobes of the prostate were prominent. Uroflowmetry showed a maximum flow of 23.1mL/s and post-void residual urine volume of 35mL. This patient has been followed up for six months.

The other patient, a 59-year old man with hypertension and a history of symptomatic benign prostatic hyperplasia was submitted to transurethral resection of the prostate in 2010 and again in 2015. One year later, complaints of a weak urine stream and having to strain to void resulted in a diagnosis of urethral stricture. He underwent internal urethrotomy, which was followed by a temporary improvement in his symptoms; however, stenosis recurred after a year. Voiding cystourethrogram revealed stricture of the bulbar urethra (Figure-2B). Uroflowmetry showed a flattened curve with a maximum flow of 6mL/s. He was submitted to urethroplasty using the technique described in this paper. After surgery, the patient had a normal urine stream. Postoperative uroflowmetry showed a maximum flow of 16.5mL/s and a bell-shaped curve. The patient has now been followed up for four months.

## DISCUSSION

Urethral stenosis differs according to its etiology, extent, site, depth and density. All these factors are relevant to the management of this pathology, determining the most appropriate approach in each case (7). The use of urethral dilatation, internal urethrotomy and primary reconstruction are treatment options, however, there are limitations when the stenosis is complex and extensive. In such cases, the use of flaps and grafts has been proposed. These can be of different origins, including lingual mucosa, labial mucosa, postauricular mucosa, etc. In 1993 in Egypt, El-Kasaby et al. described the use of a buccal mucosa patch graft in urethroplasty (8), giving rise to the development of various different techniques. In 1996, Morey et al. (9) described ventral on-lay oral mucosa urethroplasty and proposed an improvement to the technique used to harvest buccal mucosa using two teams working simultaneously. Two years later, Barbagli et al. (6) described the application of a dorsal on-lay graft with preservation of the ventral surface. Asopa et al. also described the use of a dorsal on-lay graft (10). More recently, dorsal grafts have been used in less extensive dissections of the urethra, as described by Kulkarni et al. (5).

In selected patients, double graft urethroplasty has also been used for more severe forms of stenosis, with little or no lumen for this purpose. Several variations in techniques have been used (4, 11–13). Palminteri et al. described a technique in which a combined dorsal plus ventral double buccal mucosa graft was used in the urethra. Despite encouraging results, in our opinion there are two disadvantages with that technique. The first is the need for two incisions in the urethra, one ventral and the other dorsal. The second disadvantage is that the ventral graft has little support for its fixation, since it is not fixed to the corpus spongiosum.

The technique described here offers the considerable advantage of increasing the lumen, both in the dorsal and ventral parts, using only a urethral incision on the dorsal surface. The longitudinal relaxing incision in the urethra is similar to the transurethral incision for hypospadias, as described by Snodgrass et al. (14). This incision allows the urethral diameter to be increased, with the fault in the midline being filled by the buccal mucosa inlay graft. Preservation of the corpus spongiosum allows the graft to be fixed in the same way as dorsal grafts are fixed onto the corpus cavernosum, using multiple, separate sutures. The rest of the dorsal graft technique is similar to that described by Barbagli et al. (6). We believe that it can be used, not only for fine-caliber strictures, but also for any type of stenosis. The major limitations of this preliminary study, however, are the short follow-up and the limited number of cases.

## CONCLUSION

A double-graft urethroplasty with buccal mucosa using a longitudinal urethral incision preserving the corpus spongiosum and inlay graft proved a viable option, with good results in the postoperative follow-up of these two patients. Further studies involving larger numbers of patients with control groups and long-term follow-up are required to evaluate the effectiveness of this method.
